# Circulating periostin in relation to insulin resistance and nonalcoholic fatty liver disease among overweight and obese subjects

**DOI:** 10.1038/srep37886

**Published:** 2016-11-25

**Authors:** Zhen Yang, Hongmei Zhang, Yixin Niu, Weiwei Zhang, Lingfei Zhu, Xiaoyong Li, Shuai Lu, Jiangao Fan, Xiaoying Li, Guang Ning, Li Qin, Qing Su

**Affiliations:** 1 Department of Endocrinology, Xinhua Hospital, Shanghai Jiaotong University School of Medicine, Shanghai, China; 2 Department of Endocrinology, Xinhua Hospital Chongming Branch, Shanghai Jiaotong University School of Medicine, Shanghai, China; 3 Department of Gastroenterology, Shanghai Key Laboratory of Children’s Digestion and Nutrition, Xinhua Hospital, Shanghai Jiaotong University School of Medicine, Shanghai, China; 4 Shanghai Institute of Endocrinology and Metabolism, Department of Endocrine and Metabolic Diseases, Shanghai Clinical Center for Endocrine and Metabolic Diseases, Ruijin Hospital, Shanghai Jiao Tong University School of Medicine, Shanghai, China; 5 The Key Laboratory of Endocrine Tumors and the Division of Endocrine and Metabolic Diseases, E-Institute of Shanghai Universities, Shanghai, China

## Abstract

Recent study showed periostin play a pivotal role in abnormal liver triglyceride (TG) accumulation and in the development of obesity-related liver fat accumulation. However, little is known regarding whether periostin plays a key role in the heightened prevalence of NAFLD and other metabolic phenotypes among large-scale populations. A cross-sectional sample of 8850 subjects aged 40 yr or older from China were evaluated in this study. Serum periostin was measured by ELISA methods. The diagnosis of NAFLD by liver ultrasonic examination. Among overweight and obese subjects, NAFLD subjects had higher serum periostin levels than those without NAFLD (126.75 ng/ml vs. 75.96 ng/ml, p < 0.001). Periostin was associated with a higher risk for NAFLD (OR 1.75 for each SD increase in periostin, 95% CI 1.04–3.37, p < 0.001) among overweight and obese subjects after confounder adjustment. Furthermore, periostin levels among overweight and obese subjects were correlated with aspartate aminotransferase (r = 0.102, p = 0.004), alanine aminotransferase (r = 0.108, p = 0.003), waist circumference (r = 0.111, p = 0.002), homeostasis model assessment index-insulin resistance (r = 0.154, p < 0.001) and fasting plasma insulin (r = 0.098, p = 0.006), TG (r = 0.117, p = 0.001). Elevated circulating periostin level was associated with an increased risk of having NAFLD and insulin resistance among overweight and obese individuals.

Obesity is tightly associated with an increased risk of NAFLD^1^, epidemiological data indicated that with up to 95% of obese persons likely to have NAFLD, with most cases unrecognized^2^. Aberrant triglyceride accumulation is considered as the hallmark of NAFLD^3^. Metabolic syndrome and obesity is closely related to this dysregulated hepatic lipid accumulation^4^.

Periostin is a secreted cell adhesion protein of fasciclin family^5^. Previous studies have demonstrated that periostin play an important role in the development of multiple tumors, tooth and bone formation^6^,^7^,^8^. Recently, Lu *et al*. revealed that periostin is evidently upregulated in obese rodents and humans livers tissue^9^. Periostin is involved in abnormal liver fat homeostasis in obesity^9^. Periostin could mediates obesity-induced hepatosteatosis by promotes hepatic triglyceride accumulation by downregulation of PPARa^9^. Liver tissue Periostin levels were remarkably increased in NAFLD subjects and well correlated with liver triglyceride content^9^. In addition, increased serum periostin concentrations were also observed in human NAFLD subjects^9^, indicated that periostin may be a promising extracellular diagnosis biomarker of obesity-induced hepatosteatosis^10^. Very recently, Li *et al*. demonstrated that periostin is highly expressed in methionine-choline-deficient (MCD) diet-induced NASH mice^11^. Moreover, the degree of inflammation, steatosis and fibrosis in *Postn*^−/−^ mice dramatically lower than wild type mice after administered the MCD diet^11^. Furthermore, previous studies have also confirmed that several hepatokines which secreted by liver could involved in regulate systemic and liver lipid and glucose metabolism^12^,^13^,^14^. Taken together, these findings indicated that periostin could also serve as a novel hepatokine to regulate hepatic fat metabolism.

Furthermore, *Postn*^−/−^ mice showed specifically impaired pancreatic regeneration in the islet β-cell^15^. Increased insulin expressed and a markedly improvement in glucose homeostasis was also observed after administered periostin via the bile duct^15^. Therefore, periostin might also play an essential role in pancreas regeneration and is capable of inducing β-cell regeneration. Nevertheless, epidemiological studies investigating the relation between circulating periostin level and NAFLD and other metabolic phenotype were not available.

Thus, the purpose of this study is to examine the association between serum periostin levels and NAFLD as well as other metabolic phenotypes in Chinese people.

## Results

The biochemical and clinical parameters stratified by NAFLD were shown for lean, and overweight and obese are presented in Table 1. In lean groups, subjects with NAFLD were older, more central obesity, and had higher total cholesterol, triglycerides, LDL-c, fasting plasma glucose, 2 h plasma glucose, fasting serum insulin, HOMAIR, CRP, and liver enzymes and had lower adiponectin, HDL-c and eGFR (all P < 0.01). Similarly, in overweight and obese groups, participants with NAFLD were older, and had higher BMI, WC, total cholesterol, triglycerides, LDL-c, fasting plasma glucose, 2 h plasma glucose, fasting serum insulin, HOMAIR, CRP, and liver enzymes and had lower adiponectin, HDL-c and eGFR (all P < 0.01).

Circulating periostin was significantly and positively correlated with WC, fasting insulin, AST and HOMA-IR among all subjects (all p < 0.05). Especially in overweight and obese, circulating periostin levels were positively correlated with WC, fasting serum insulin, triglycerides, AST, ALT, GGT and HOMA-IR (all p < 0.05) (Table 2).

**Figure 1:**
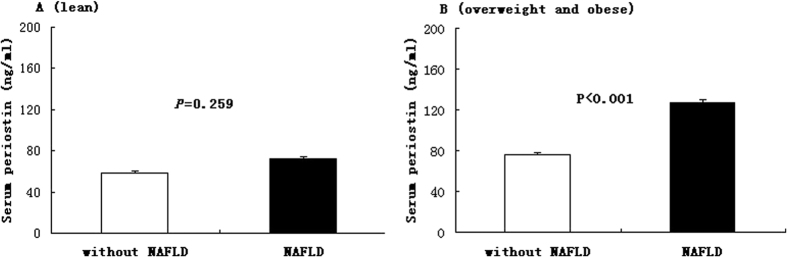
Serum periostin in without NAFLD and NAFLD subjects according to obesity status (A for lean and B for overweight and obese). Data are shown as means ± SE after adjustment for age and sex.

Figure 1 showed the circulating periostin levels in without NAFLD and NAFLD subjects according to obesity status. Among overweight and obese subjects, NAFLD patients showed circulating periostin value higher than their counterpart non-NAFLD subjects (126.75 ± 21.37 ng/ml vs. 75.96 ± 17.92 ng/ml, p < 0.001), whereas lean subjects did not show any significant difference in periostin levels based on NAFLD (72.65 ± 18.15 ng/ml vs. 58.59 ± 16.36 ng/ml, p = 0.259).

Table 3 showed subjects with 1-SD increase had higher OR for the risk of NAFLD among overweight and obese subjects (OR 1.75; 95% CI 1.04–3.37; P < 0.001) after adjustment for gender, age, smoking, eGFR, WC, BMI, HOMA-IR and lipid profiles. However, the significant associations were not detected in lean subjects.

## Discussion

In this study, we demonstrated that higher periostin levels were significantly associated with increased risk of having NAFLD among overweight and obese subjects. Moreover, increased circulating periostin levels were also significantly correlated with increased insulin resistance, particularly among overweight and obese subjects.

Abnormal triglyceride accumulation in liver is recognized as the hallmark of NAFLD. Hepatic lipid accumulation in NAFLD impairs insulin signaling that contributes to abnormal hepatic metabolism^16^. This dysregulated liver fat accumulation is closely related to obesity, diabetes and metabolic syndrome^3^,^16^. In humans, obesity is strongly associated with hepatosteatosis and NASH pathogenesis^17^. We found that circulating periostin is notable increased in subjects with NAFLD among overweight and obese individuals. Regression analysis further indicated that elevated circulating periostin was independently associated with higher presence of NAFLD among overweight and obese subjects. Although the accurate mechanism for explanation these phenomenon remains unknown. Lu *et al*. has showed that periostin as a potent regulator of hepatic lipid accumulation via activation of the JNK/c-Jun pathway, which prevented expression of PPARα, in obesity mouse primary hepatocytes^9^. In addition, increased circulating periostin levels were also observed in obesity human NAFLD subjects, although there is no significant association between circulating periostin concentrations and liver triglycerides content^9^. Our study also observed the significantly association of GGT, AST and ALT with periostin. It has well established that GGT, AST and ALT are widely accepted noninvasive biomarkers of liver injury^16^. Additionally, we found that periostin was correlated with TG in overweight and obese subjects. Accordingly, all these findings suggest that periostin could also serve as a hepatokine in process of regulation of hepatic TG metabolism, although the underlying mechanisms need further extensive exploration^18^.

Previous study also suggests that ChREBP, a transcription factor which plays an important role in the induction of glucose-regulated genes in liver^19^,^20^, could trigger the expression of periostin in liver cell by glucose^9^. We found circulating periostin was significantly positively correlated with waist circumference rather than BMI. However, the periostin levels showed no significant correlated with the FPG and 2 h PG. These results suggest that the change in periostin levels might be associated with an alteration in body composition, but not with a simple change in body weight and plasma glucose. Further experiments are required to elucidate the relationship of visceral fat and serum periostin concentrations.

We observed a significant positive correlation between circulating periostin levels and fasting plasma insulin, and insulin resistance assessed by HOMA-IR in overweight and obese individuals, but not in normal weight participants. Moreover, Lu *et al*.^9^ reported obese high-fat diet-fed and ob/ob mice have higher circulating periostin levels. Although the underlying mechanism is unclear, these findings provided novel insights into the relationship of adipogenesis and periostin secretion. Certainly, further experiments are required to elucidate the interaction of periostin and insulin resistance. In addition, despite it has been well documented that obesity cause insulin resistance, which is involved in the pathogenesis of NAFLD, here we found that the participants with a 1-SD increase in circulating periostin showed a 1.75 times risk for NAFLD, no matter the degree of insulin resistance, indicating that alone an increased periostin concentrations could augment the NAFLD phenotype by the mechanisms differ from insulin resistance.

As we best known, this is the first study specifically aimed at exploring the relationship between circulating periostin concentrations and NAFLD in a large sample population. The confounding effects have been minimized because most potential covariates were carefully adjusted. However, several limitations should be addressed. The cause-effect inference can not be drawn because of the cross-sectional nature of the current study. In addition, liver biopsies, the gold standard for diagnosed fatty liver, were not available in this study. The NAFLD diagnosis was based on ultrasound imaging, which means that NAFLD patients in our study were in at least moderate stage of the disease. Therefore, we failed to assess the correlation between circulating periostin and mild-stage NAFLD in this study. However, given the several advantages of ultrasound imaging, including portability, low cost, and simplicity of use, made it further applicable and acceptable for investigating the incidence, prevalence, and risk factors of NAFLD, this technique is the most widely used noninvasive method to detect hepatic lipid accumulation in epidemiological investigations and clinical practice.

In summary, our study demonstrated that increased circulating periostin was markedly associated with an increased presence of NAFLD in Chinese overweight and obese subjects. Further experimental and longitudinal investigations are expected to determine the role of periostin in the development of NAFLD.

## Methods

### Study participants and design

In 2011, China lunched a national survey of Risk Evaluation of cAncers in Chinese diabeTic Individuals: a lONgitudinal (REACTION) study, which was conducted among 259,657 adults, aged 40 years and older in 25 communities across mainland China, from 2011 to 2012^21^. The data presented in this article are based on the baseline survey of subsamples from Chongming District, Shanghai, China. There were 9930 participants who had complete information about age; sex; smoking and alcohol consumption habits and medical history, BMI, and a hepatic ultrasonic examination. Main exclusion criteria: (1) serious liver diseases (including malignancy, hepatitis, liver cirrhosis); (2) alcohol consumption greater than 70 g/wk for women and 140 g/wk for men. Thus, total number of participants who eventually included in this analysis was 8850. The study protocol was approved by the Ethics Committee of Xinhua Hospital Affiliated to Shanghai Jiaotong University School of Medicine, and all studies were carried out in accordance with the approved guidelines. Written informed consent was obtained from all the participants.

### Data collection

Age, gender, medical hospital and life habits were collected by trained physicians. The smoking habit was defined as never or current (smoking regularly in the past 6 months). The history of drinking was also collected.

Overnight fasting and 2 h OGTT blood samples were collected for analysis. The details of anthropometric measurements including height, weight, waist circumference, hip circumference were carried by trained medical worker. Blood pressure was obtained with an automated electronic device (OMRON Model1 Plus; Omron Company, Kyoto, Japan). Obesity was defined according to the standard for Chinese individuals: subjects with BMI < 24.0 kg/m^2^ defined as normal weight, BMI ≥24.0 kg/m^2^ defined as overweight or obesity^22^.

### Laboratory methods

All subjects were assessed after overnight fasting for at least 10 h, Overnight fasting and 2 h OGTT blood samples were collected in tubes containing EDTA and were centrifuged at 4 °C and stored at −80 °C until analysis. The fasting glucose, glucose 2 h after oral glucose tolerance test, alanine aminotransferase (ALT), aspartate aminotransferase (AST), and γ-glutamyltranspeptidase (GGT), creatinine, total cholesterol (TC), triglycerides (TG), low-density lipoprotein (LDL) cholesterol and high-density lipoprotein (HDL) cholesterol were measured on an automatic analyzer (Hitachi 7080; Tokyo, Japan). Hemoglobin A1c was determined by HPLC method (BIO-RAD, D10, CA). Circulating C-reactive protein and Interleukin-6 (IL-6) was measured by ELISA kit (R&D Systems, Minneapolis, MN). Fasting insulin was determined by RIA (Linco Research, St. Charles, MO). Insulin resistance was measured by the homeostasis model of assessment for insulin resistance (HOMA-IR)^23^. The estimated glomerular filtration rate (eGFR) was measured by abbreviated Modification of Diet in Renal Disease formula recalibrated for Chinese^24^.

### Measurement of circulating adiponectin, CRP and periostin concentration

The circulating adiponectin, CRP and periostin were duplicated measured by ELISA kit (DY1065, DY1707, and DY3548; R&D Systems, Minneapolis, MN) according to manufacturer’s recommendation.

### Liver ultrasound evaluation

Abdominal ultrasound examination was performed after overnight fasting, by two expert physician, who blinded to the clinical and biochemical parameter of subjects, with a 3.5-MHz convex probe and a high-resolution B-mode scanner (Esaote Biomedica SpA, Italy). Diagnosis of fatty liver based on increased hepatic echogenicity compared to renal cortex^25^,^26^.

### Statistical analysis

Results were expressed as means ± SD for normally distributed variables and as median (interquartile range) for skewed distribution variables. The skewed distribution variables were log transformed to approximate normality before analysis. Comparisons between the continuous variables and frequencies were performed using the Mann-Whitney U test, 2-sample t test and χ^2^ tests, respectively. Spearman correlation test was used to determine the association between circulating periostin concentrations and the study variables. To investigate the associations between circulating periostin concentrations and NAFLD, the multivariate adjusted logistic regression analyses were performed to assess the OR for the risk of NAFLD. Statistical analyses were performed using the statistical software package SPSS, version 13.0 for Windows (SPSS Inc., IL). A two-sided P value < 0.05 was considered to be significant.

## Additional Information

**How to cite this article**: Yang, Z. *et al*. Circulating periostin in relation to insulin resistance and nonalcoholic fatty liver disease among overweight and obese subjects. *Sci. Rep.* **6**, 37886; doi: 10.1038/srep37886 (2016).

**Publisher’s note:** Springer Nature remains neutral with regard to jurisdictional claims in published maps and institutional affiliations.

## Figures and Tables

**Table 1:** Anthropometric and metabolic characteristics of the study subjects.

	Lean (n = 4033)			Overweight and obese (n = 4817)		
	NAFLD (−)	NAFLD (+)	P value	NAFLD (−)	NAFLD (+)	P value
n (%)	3202 (79.4)	831 (20.6)		1916 (39.8)	2901 (60.2)	
Age (yr)	54.9 ± 8.2	56.3 ± 7.6	<0.0001	56.3 ± 7.9	57.2 ± 7.5	<0.0001
BMI (kg/m^2^)	21.6 ± 1.7	22.4 ± 1.4	<0.0001	26.2 ± 2.2	27.6 ± 6.6	<0.0001
Waist circumference (cm)	76.8 ± 7.9	80.9 ± 6.7	<0.0001	88.1 ± 10.9	91.5 ± 8.6	<0.0001
TG (mmol/L)	1.08 (0.81–1.52)	1.71 (1.17–2.48)	<0.0001	1.23 (0.92–1.80)	1.76 (1.27–2.57)	<0.0001
TC (mmol/L)	4.54 ± 0.99	4.85 ± 1.06	<0.0001	4.58 ± 1.01	4.75 ± 1.05	<0.0001
LDL-c (mmol/L)	2.53 ± 0.74	2.71 ± 0.82	<0.0001	2.57 ± 0.73	2.69 ± 0.79	<0.0001
HDL-c (mmol/L)	1.31 ± 0.33	1.20 ± 0.29	<0.0001	1.24 ± 0.33	1.13 ± 0.27	<0.0001
HOMA-IR	1.37 (0.96–1.80)	2.19 (1.57–2.86)	<0.0001	1.65 (1.24–2.28)	2.61 (1.89–3.57)	<0.0001
Fasting plasma glucose (mmol/L)	5.96 ± 1.46	6.74 ± 2.24	<0.0001	6.12 ± 1.44	6.62 ± 1.83	<0.0001
2 h OGTT plasma glucose (mmol/L)	7.66 ± 3.42	9.80 ± 4.69	<0.0001	8.12 ± 3.41	9.93 ± 4.10	<0.0001
Fasting serum insulin (μU/ml)	5.51 (3.80–6.80)	7.20 (5.50–9.60)	<0.0001	6.10 (4.50–8.00)	9.00 (6.90–11.90)	<0.0001
ALT (IU/L)	11.0 (8.0–16.0)	16.0 (11.0–25.0)	<0.0001	13.0 (10.0–18.0)	17.0 (12.0–26.0)	<0.0001
AST (IU/L)	18.0 (14.0–22.0)	19.0 (16.0–25.0)	<0.0001	18.0 (15.0–23.0)	20.0 (16.0–25.0)	<0.0001
GGT (IU/L)	15.0 (11.0–23.0)	23.0 (15.0–40.0)	<0.0001	18.0 (13.0–29.0)	25.0 (17.0–41.0)	<0.0001
eGFR (ml/min per 1.73 m^2^)	126.1 (111.8–140.4)	120.6 (107.2–135.4)	<0.0001	123.1 (108.9–139.6)	121.2 (108.3–136.2)	<0.0001
CRP (μg/mL)	4.1 ± 3.5	5.6 ± 4.4	<0.0001	5.3 ± 4.1	6.4 ± 4.8	<0.0001
Adiponectin (μg/mL)	10.51 (7.69–14.07)	8.74 (5.85–12.53)	<0.0001	7.69 (5.52–10.19)	6.85 (5.19–9.88)	<0.0001
Periostin (ng/ml)	58.59 ± 16.25	76.25 ± 18.59	0.259	75.96 ± 20.15	126.75 ± 85.64	<0.0001
Current smoking, n (%)	526 (16.4)	118 (14.2)	0.064	348 (18.2)	482 (16.6)	0.088

Data are means ± SD or medians (interquartile ranges) or numbers (proportions). P values were calculated from χ^2^ tests for categorical variables and Student’s t tests for continuous variables. NAFLD, nonalcoholic fatty liver disease; BMI, body mass index; TG, triglycerides; TC, total cholesterol; LDL-c, low-density lipoprotein cholesterol; HDL-c, high-density lipoprotein cholesterol; HOMA-IR, insulin resistance index for homeostasis model assessment; AST, aspartate aminotransferase; ALT, Alanine aminotransferase; GGT, γ-glutamyltransferase; eGFR, estimated glomerular filtration rate; CRP, C-reactive protein.

**Table 2:** Correlations between periostin levels and various parameters of the study subjects.

	Lean		Overweight and obese		Total	
	r	P value	r	P value	r	P value
Age	0.026	0.459	0.035	0.322	0.004	0.940
BMI (kg/m^2^)	0.021	0.550	0.067	0.060	0.041	0.253
Waist circumference (cm)	0.033	0.361	0.111	0.002	0.082	0.022
Fasting plasma glucose (mmol/l)	0.015	0.666	0.089	0.062	0.045	0.348
2 h OGTT plasma glucose (mmol/l)	0.011	0.822	0.029	0.549	0.018	0.656
Log10 fasting plasma insulin (μU/ml)	0.094	0.015	0.098	0.006	0.088	0.017
Log10 HOMA-IR	0.014	0.762	0.154	<0.0001	0.100	0.005
TG (mmol/L)	0.067	0.060	0.117	0.001	0.064	0.073
TC (mmol/L)	0.020	0.574	0.052	0.174	0.028	0.439
LDL-c (mmol/L)	0.021	0.552	0.048	0.188	0.05	0.162
HDL-c (mmol/L)	−0.011	0.751	−0.003	0.943	−0.009	0.792
AST (IU/L)	0.028	0.436	0.102	0.004	0.078	0.029
ALT (IU/L)	0.047	0.186	0.108	0.003	0.053	0.151
GGT (IU/L)	0.030	0.524	0.085	0.019	0.039	0.276
eGFR (ml/min per 1.73 m^2^)	0.028	0.439	0.046	0.199	0.052	0.147
CRP (μg/ml)	0.021	0.550	0.056	0.115	0.031	0.383
Adiponectin (μg/mL)	−0.002	0.966	−0.008	0.822	−0.007	0.840

NAFLD, nonalcoholic fatty liver disease; BMI, body mass index; TG, triglycerides; TC, total cholesterol; LDL-c, low-density lipoprotein cholesterol; HDL-c, high-density lipoprotein cholesterol; HOMA-IR, insulin resistance index for homeostasis model assessment; AST, aspartate aminotransferase; ALT, Alanine aminotransferase; GGT, γ-glutamyltransferase; eGFR, estimated glomerular filtration rate; CRP, C-reactive protein.

**Table 3:** The risk of NAFLD associated with a 1-SD increase in serum periostin.

	Lean		Overweight and obese	
	OR (95% CI)	*P* value	OR (95% CI)	*P* value
Model 1	1.25 (0.93–1.84)	0.13	2.13 (1.16–3.77)	<0.001
Model 2	1.17 (0.81–1.77)	0.27	2.04 (1.12–3.70)	<0.001
Model 3	1.12 (0.77–1.73)	0.43	1.88 (1.07–3.56)	<0.001
Model 4	1.08 (0.75–1.68)	0.52	1.75 (1.04–3.37)	<0.001

OR, odds ratio; CI, confidence interval. We defined participants without NAFLD as 0 and those with NAFLD as 1. Model 1 was adjusted for age, sex, smoking, and eGFR. Model 2 was further adjusted for BMI and waist circumference based on model 1. Model 3 was further adjusted for serum TG, TC, HDL-c, and LDL-c based on model 2. Model 4 was further adjusted for HOMA-IR based on model 3.
